# Progression of endothelial dysfunction, atherosclerosis, and arterial stiffness in stable kidney transplant patients: a pilot study

**DOI:** 10.1186/s12872-019-01309-y

**Published:** 2020-01-08

**Authors:** Joey Junarta, Nina Hojs, Robin Ramphul, Racquel Lowe-Jones, Juan C. Kaski, Debasish Banerjee

**Affiliations:** 1 Renal and Transplantation Unit, St George’s University Hospitals, NHS Foundation Trust, London, UK; 2 Cardiology Clinical Academic Group, Molecular and Clinical Sciences Research Institute, St George’s University of London, London, UK

**Keywords:** Endothelial function, Arterial stiffness, Atherosclerosis, Kidney transplantation

## Abstract

**Background:**

Kidney transplant patients suffer from vascular abnormalities and high cardiovascular event rates, despite initial improvements post-transplantation. The nature of the progression of vascular abnormalities in the longer term is unknown. This pilot study investigated changes in vascular abnormalities over time in stable kidney transplant patients long after transplantation.

**Methods:**

Brachial artery flow-mediated dilation (FMD), nitroglycerin-mediated dilation, carotid-femoral pulse wave velocity (cf-PWV), ankle-brachial pressure index, and common carotid artery intima-media thickness (CCA-IMT) were assessed in 18 kidney transplant patients and 17 controls at baseline and 3–6 months after.

**Results:**

There was no difference in age (51 ± 13 vs. 46 ± 11; *P* = 0.19), body mass index (26 ± 5 vs. 25 ± 3; *P* = 0.49), serum cholesterol (4.54 ± 0.96 vs. 5.14 ± 1.13; *P =* 0.10), systolic blood pressure (BP) (132 ± 12 vs. 126 ± 12; *P* = 0.13), diastolic BP (82 ± 9 vs. 77 ± 8; *P* = 0.10), or diabetes status (3 vs. 0; *P* = 0.08) between transplant patients and controls. No difference existed in vascular markers between patients and controls at baseline. In transplant patients, FMD decreased (− 1.52 ± 2.74; *P* = 0.03), cf-PWV increased (0.62 ± 1.06; *P* = 0.03), and CCA-IMT increased (0.35 ± 0.53; *P* = 0.02). No changes were observed in controls.

**Conclusion:**

Markers of vascular structure and function worsen in the post-transplant period on long-term follow-up, which may explain the continued high cardiovascular event rates in this population.

## Background

Patients with chronic kidney disease (CKD) have a higher burden of cardiovascular disease (CVD), including kidney transplant recipients [1]. Although cardiovascular risk factors improve in the immediate perioperative period, the long-term risk remains high [2, 3]. CVD is the commonest cause of death in transplant patients with a surviving graft, more so than infection or malignancy [4].

Endothelial dysfunction, arterial stiffness, and accelerated atherosclerosis are common in stable kidney transplant patients and may contribute to the high cardiovascular event rate [3, 5, 6]. Endothelial dysfunction, a prerequisite to atherosclerosis, encompasses numerous maladaptive alterations adversely affecting vascular tone, haemostasis, and inflammatory processes within the arterial wall [7]. Both traditional and non-traditional risk factors in transplant patients can induce endothelial dysfunction [3, 8]. Calcification of the arterial wall is common in transplant patients and contributes to vascular stiffness [3, 9].

The nature of the changes in these vascular abnormalities in kidney transplant recipients is unknown. Previous studies mostly examined changes in the vascular properties of transplant patients pre-transplantation and immediately post-transplantation. They were not examined in stable transplant recipients long after transplantation. If these changes are adverse, they may be the result of novel risk factors post-transplantation and may be a cause of the high cardiovascular event rate. This pilot study investigated the changes in endothelial dysfunction, arterial stiffness, and atherosclerosis in stable kidney transplant recipients long into the post-transplant period.

## Methods

### Study population and design

**Fig. 1:**
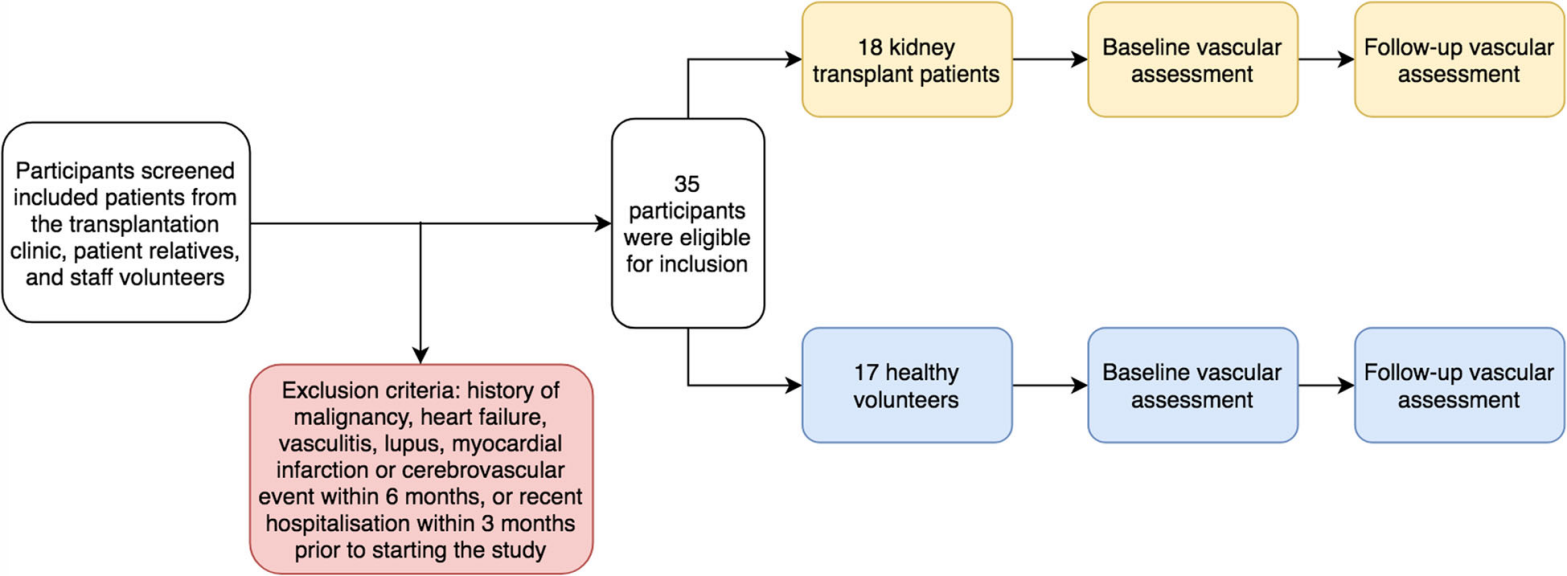
Flowchart of study design

Participants included patients recruited from the transplantation clinic, patient relatives, and staff volunteers. Patients were eligible if they were between 18 to 80 years of age with stable kidney function for ≥3 months (estimated glomerular filtration rate [eGFR] change < 5 ml/min/1.73m^2^) and have been transplanted for ≥6 months with or without previous dialysis. Exclusion criteria included a history of malignancy, heart failure, vasculitis, lupus, myocardial infarction or cerebrovascular event within 6 months or recent hospitalisation within 3 months prior to starting the study. The study was approved by the London South East Research Ethics Committee. All participants gave informed consent prior to their inclusion in the study. Vascular parameters were measured in a quiet vascular laboratory with a controlled temperature of 22–24 °C at recruitment (baseline visit) and 3–6 months after recruitment (second visit). Figure 1 demonstrates a flow chart of the study design. Vascular parameters measured include brachial flow-mediated dilation (FMD), nitroglycerin-mediated dilation (NMD), carotid-femoral pulse wave velocity (cf-PWV), and common carotid artery intima-media thickness (CCA-IMT). Participants were required to withhold taking anti-hypertensive medications 24 h before vascular assessment to prevent interference. In the absence of local data in transplant patients we used our previous data in CKD patients for power calculation. For pre-dialysis CKD patients in our previous study the FMD was 3.1 ± 3.3% [10]. To demonstrate a clinically significant difference of 60% with α = 0.05 and β = 0.80, 27 patients are needed. For CKD patients (pre-dialysis and post-kidney transplantation) in our previous study the CCA-IMT was 0.77 ± 0.17 [3]. To demonstrate a clinically significant difference of 10% over 1 year with α = 0.05 and β = 0.80, 36 patients are needed. Assuming a dropout rate of 10%, 40 patients are needed. The main aim of this pilot study was to recruit enough patients and volunteers to demonstrate a difference in change of endothelial function.

### Clinical characteristics

A standardised data form was used by researchers to record participants’ medical histories, including to obtain systematic information on the presence of cardiovascular risk factors. Weight, height, waist and hip circumference, and two blood pressure (BP) measurements were taken. Body mass index (BMI) and waist to hip ratio (WHR) were calculated.

### Flow-mediated dilation, nitroglycerin-mediated dilation, common carotid artery intima-media thickness, and carotid-femoral pulse wave velocity

Brachial FMD, NMD, and CCA-IMT were performed as described in previous studies by our group [3]. Cf-PWV was performed as described before [11]. Brachial FMD and NMD were assessed using a standard High-Definition-Imaging-3000 ultrasound system (ATL, Bothell, WA, USA) equipped with a 12–5 MHz linear-array transducer. Cf-PWV was assessed using the SphygmoCor2000 system (AtCor Medical, Sydney, Australia). CCA-IMT was measured using a Vivid7 ultrasound machine (GE Healthcare, Wauwatosa, WI, USA) equipped with a linear 13–5 MHz transducer. The overall mean (standard deviation) intra-operator variability of FMD within our department is 0.90 (0.48)% [12].

### Ankle-brachial pressure index

Ankle-brachial pressure index (ABPI) was assessed with a continuous wave Doppler instrument (8–10 MHz). The ABPI of each leg was calculated by dividing the highest systolic BP in the desired leg between the measurement of the dorsalis pedis and posterior tibial artery divided by the highest systolic BP between the two arms. The mean ABPI value for each participant was derived from the average between the ABPI of the right and left leg.

### Laboratory measurements

Blood and urine samples were collected for biochemical analysis at the South West London Pathology (SWLP) services. The internal quality assurance of the SWLP service is guaranteed by strict adherence to analytical protocols and a comprehensive internal quality control programme. The SWLP service subscribes to all national external quality assessment schemes for the tests they provide and their laboratories are also registered with the Clinical Pathology Accreditation of the United Kingdom Accreditation Service, which ensures that the services meet the stringent requirements expected from a pathology service. eGFR was calculated using the Chronic Kidney Disease Epidemiology Collaboration (CKD-EPI) 2009 equation [13].

### Statistical analyses

Continuous variables were analysed with an independent samples t-test, categorical variables using a chi-squared test, and within-group comparisons using a paired t-test. Bivariate correlations between continuous variables were calculated using Pearson’s correlation coefficient (r). Point-Biserial correlation (r_pb_) was used to determine correlations between binary and continuous variables. A two-sided *P*-value of < 0.05 was used to determine statistical significance. Analyses were performed using SPSS version 25.0 (SPSS Inc., Chicago, IL, USA).

## Results

Eighteen kidney transplant patients and 17 healthy volunteers were enrolled in the study. The mean number of days between the first and second visit for transplant patients was 161 ± 36 days and 185 ± 52 days for controls. The mean duration on dialysis (haemodialysis and/or peritoneal dialysis) before transplantation was 29 ± 14 months (median 29, interquartile range [IQR] 28). The median time since transplantation at recruitment was 86 months with an IQR of 123 months. Transplant patients were taking up to 3 of 6 different immunosuppressants at recruitment. The immunosuppressants used were Tacrolimus (89%), Mycophenolate Mofetil (39%), Prednisolone (44%), Azathioprine (22%) and Sirolimus (6%). The aetiology of CKD in our transplant patients were the following: autosomal dominant polycystic kidney disease (4), IgA nephropathy (4), hypertension (2), pyelonephritis (1), focal segmental glomerulosclerosis (1), gout nephropathy (1), and type 2 diabetes mellitus (1). Four transplant patients had an unclear aetiology to their CKD.

### Baseline parameters in study participants

Table 1 shows the baseline clinical characteristics and circulating biomarkers in study participants. The kidney transplant patient group had reduced eGFR, more dyslipidaemics, and elevated parathyroid hormone and N-terminal-pro-brain natriuretic peptide levels. Table 2 shows the baseline vascular structure and function of participants. No difference existed between transplant patients and controls.

**Table 1:** Clinical characteristics and circulating biomarkers in study participants

Parameter	Healthy controls (*n* = 17)			Kidney transplant patients (*n* = 18)			Between-group comparison
	Baseline	Second visit	*P*-value	Baseline	Second visit	*P*-value	Baseline results *P*-value
Age (years)	45.82 ± 10.85	NA	NA	51.28 ± 13.29	NA	NA	0.19
Gender M/F	5/12	NA	NA	9/9	NA	NA	0.21
Body mass index (kg/m^2^)	24.59 ± 2.59	25.00 ± 2.66	0.09	25.56 ± 5.18	24.03 ± 7.63	0.36	0.49
Waist/Hip	0.82 ± 0.07	0.80 ± 0.06	0.10	0.86 ± 0.10	0.93 ± 0.14	0.07	0.16
Systolic blood pressure (mmHg)	125.53 ± 12.39	119.76 ± 10.62	0.02	131.94 ± 11.79	130.44 ± 10.20	0.61	0.13
Diastolic blood pressure (mmHg)	77.24 ± 7.61	76.24 ± 8.70	0.64	82.17 ± 9.22	80.56 ± 7.45	0.40	0.10
Smoking status past/present/never	0/6/11	NA	NA	0/4/14	NA	NA	0.39
Diabetes mellitus	0	NA	NA	3	NA	NA	0.08
History of IHD	0	NA	NA	0	NA	NA	NA
Dyslipidaemia	3	NA	NA	10	NA	NA	0.02
Haemoglobin (g/L)	134.29 ± 11.86	138.71 ± 9.31	0.02	135.33 ± 18.01	135.72 ± 15.11	0.85	0.84
Albumin (g/L)	39.35 ± 2.47	40.12 ± 2.32	0.19	38.11 ± 2.72	38.83 ± 2.96	0.34	0.17
Urea (mmol/L)	4.26 ± 0.95	4.60 ± 1.11	0.16	7.31 ± 2.15	7.83 ± 2.67	0.05	< 0.01
Creatinine (mcmol/L)	69.65 ± 16.63	69.65 ± 15.66	1.00	104.39 ± 33.68	108.94 ± 31.53	0.14	< 0.01
eGFR (mL/min/1.73m^2^)	97.59 ± 15.59	97.29 ± 15.55	0.83	67.61 ± 20.25	61.56 ± 19.70	0.02	< 0.01
Total cholesterol (mmol/L)	5.14 ± 1.13	5.34 ± 1.21	0.16	4.54 ± 0.96	4.84 ± 1.45	0.30	0.10
HDL (mmol/L)	1.71 ± 0.56	1.75 ± 0.62	0.51	1.69 ± 0.55	1.73 ± 0.58	0.30	0.92
LDL (mmol/L)	3.24 ± 1.71	3.07 ± 0.89	0.63	2.29 ± 0.85	2.56 ± 1.25	0.29	0.04
Total cholesterol/HDL	3.29 ± 1.27	3.32 ± 1.26	0.78	3.00 ± 1.28	3.03 ± 1.18	0.84	0.50
Non-HDL (mmol/L)	3.44 ± 1.04	3.60 ± 1.04	0.09	2.85 ± 1.06	3.11 ± 1.37	0.34	0.11
Triglyceride (mmol/L)	1.05 ± 0.58	1.05 ± 0.70	0.97	1.24 ± 0.72	1.32 ± 0.74	0.55	0.56
Glucose (mmol/L)	4.71 ± 0.39	4.82 ± 0.40	0.35	5.74 ± 1.03	5.69 ± 1.34	0.80	< 0.01
Corrected calcium (mmol/L)	2.35 ± 0.05	2.39 ± 0.07	0.03	2.46 ± 0.08	2.46 ± 0.07	0.86	< 0.01
Inorganic phosphate (mmol/L)	1.11 ± 0.13	1.17 ± 0.14	0.05	0.99 ± 0.19	1.04 ± 0.19	0.14	0.03
PTH (pmol/L)	4.98 ± 1.57	4.53 ± 1.09	0.19	8.38 ± 3.68	9.42 ± 6.46	0.31	< 0.01
Vitamin D (nmol/L)	43.65 ± 22.03	67.41 ± 30.41	< 0.01	57.56 ± 25.21	66.50 ± 27.04	0.04	0.09
Troponin T (ng/L)	2.35 ± 1.46	2.76 ± 1.72	0.13	8.33 ± 4.94	8.39 ± 4.85	0.91	< 0.01
NT-pro-BNP (ng/L)	43.65 ± 25.57	40.41 ± 40.53	0.62	190.72 ± 172.10	189.44 ± 181.07	0.96	< 0.01
hsCRP (mgl/L)	1.90 ± 3.51	1.19 ± 1.64	0.45	2.58 ± 2.95	1.66 ± 1.68	0.19	0.54
Iron (mcmol/L)	18.75 ± 6.40	21.56 ± 7.72	0.06	15.50 ± 5.87	17.44 ± 6.12	0.26	0.20
Transferrin (g/L)	2.82 ± 0.32	2.83 ± 0.36	0.78	2.18 ± 0.32	2.25 ± 0.37	0.17	0.23
Ferritin (mcmol/L)	82.69 ± 78.98	87.69 ± 73.04	0.55	452.61 ± 1179.15	291.72 ± 579.02	0.28	< 0.01
Transferrin saturation (%)	27.50 ± 11.27	31.19 ± 12.58	0.08	29.44 ± 13.17	31.61 ± 11.37	0.48	0.54
ACR (mg/mmol)	0.49 ± 1.35	0.34 ± 0.78	0.76	5.53 ± 5.46	5.58 ± 7.20	0.97	< 0.01
PCR (mg/mmol)	11.12 ± 5.13	11.35 ± 3.82	0.87	15.58 ± 8.89	17.20 ± 10.83	0.36	0.05

Legend: *n* number of participants, *NA* not applicable, *M/F* male/female, *IHD* ischaemic heart disease, *eGFR* estimated glomerular filtration rate, *HDL* high-density lipoprotein, *LDL* low-density lipoprotein, *PTH* parathyroid hormone, *NT*-*pro*-*BNP* N-terminal-pro-brain natriuretic peptide, *hsCRP* highly sensitive C-reactive protein, *ACR* albumin to creatinine ratio, *PCR* protein to creatinine ratio

Data presented as mean ± standard deviation

**Table 2:** Changes in cardiovascular structure and function from baseline to second visit

Parameter	Healthy controls (*n* = 17)			Kidney transplant patients (*n* = 18)			Between-group comparison
	Baseline	Second visit	*P*-value	Baseline	Second visit	*P*-value	Baseline results *P*-value
Brachial FMD (%)	4.63 ± 3.02	3.51 ± 2.73	0.33	4.34 ± 3.45	2.82 ± 2.18	0.03	0.79
Brachial NMD (%)	16.00 ± 5.47	17.17 ± 5.18	0.31	15.15 ± 6.08	15.74 ± 3.88	0.68	0.67
Cf-PWV (m/s)	6.96 ± 1.26	7.17 ± 1.50	0.51	7.83 ± 1.76	8.44 ± 2.28	0.03	0.10
Mean ABPI	1.18 ± 0.08	1.21 ± 0.11	0.43	1.27 ± 0.15	1.23 ± 0.14	0.34	0.47
Mean CCA-IMT (mm)	5.54 ± 1.08	5.73 ± 1.34	0.22	5.73 ± 0.95	6.07 ± 0.98	0.02	0.05

Legend: *n* number of participants, *FMD* flow-mediated dilation, *NMD* nitroglycerin-mediated dilation, *Cf-PWV* carotid-femoral pulse wave velocity, *ABPI* ankle-brachial pressure index, *CCA-IMT* common carotid intima-media thickness

Data presented as mean ± SD

### Changes in clinical, circulating biomarkers, and in cardiovascular structure and function in study participants

Table 1 shows changes in clinical characteristics and circulating biomarkers in participants. In controls, systolic BP (SBP) decreased, while vitamin D and corrected calcium levels increased. In transplant patients, vitamin D levels also increased while eGFR declined. Vitamin D levels improved presumably because participants were able to request their laboratory results and may have started vitamin D therapy at their own initiative or in guidance with a clinician. Vascular structure and function did not change in controls (Table 2). In transplant patients, brachial FMD decreased (− 1.52 ± 2.74; *P* = 0.03), while cf-PWV (0.62 ± 1.06; *P* = 0.03) and CCA-IMT increased (0.35 ± 0.53; *P* = 0.02) (Table 2).

### Association of changes in FMD, cf-PWV, and CIMT in kidney transplant patients

No significant correlation existed between the decline in eGFR and changes in FMD (*r* = 0.21; *P* = 0.42), cf-PWV (*r* = 0.30; *P* = 0.23), or CIMT (*r* = 0.37; *P* = 0.15). Changes in FMD was associated with baseline haemoglobin (*r* = 0.52, *P* = 0.03), corrected calcium (*r* = 0.61, *P* = 0.01), and transferrin (*r* = 0.53, *P* = 0.03). Changes in FMD were not associated with age (*r* = 0.43; *P* = 0.08), BMI (*r* = 0.45; *P* = 0.05), WHR (*r* = 0.31; *P* = 0.21), gender (r_pb_ = − 0.18; *P* = 0.48), DM (r_pb_ = 0.18; *P* = 0.47), SBP (*r* = − 0.32; *P* = 0.07), diastolic BP (DBP) (*r* = − 0.03; *P* = 0.89), or smoking (*r* = 0.07, *P* = 0.79). Changes in FMD were not associated with any other lab values in Table 1 (*P* > 0.05). Changes in PWV was associated with baseline transferrin (*r* = 0.52, *P* = 0.03). Changes in cf-PWV were not associated with age (*r* = 0.18; *P* = 0.49), BMI (*r* = 0.27; *P* = 0.29), WHR (*r* = 0.14; *P* = 0.57), gender (r_pb_ = 0.16; *P* = 0.54), DM (r_pb_ = − 0.14; *P* = 0.59), SBP (*r* = 0.30; *P* = 0.08), or DBP (*r* = 0.08; *P* = 0.64). Changes in cf-PWV were not associated with any other lab values in Table 1 (*P* > 0.05). Changes in CCA-IMT was associated with baseline phosphate (*r* = 0.67, *P* < 0.01). Changes in CCA-IMT were not associated with age (*r* = − 0.10; *P* = 0.71), BMI (*r* = − 0.08; *P* = 0.78), WHR (*r* = − 0.29; *P* = 0.26), gender (r_pb_ = 0.14; *P* = 0.59), DM (r_pb_ = − 0.25; *P* = 0.34), SBP (*r* = 0.23; *P* = 0.19), or DBP (*r* = 0.04; *P* = 0.81). Changes in CCA-IMT were not associated with any other lab values in Table 1 (*P* > 0.05).

## Discussion

This study shows the worsening of vascular structure and function in stable kidney transplant patients, where FMD decreased while cf-PWV and CCA-IMT increased. Change in eGFR was not associated with changes in FMD, CCA-IMT, or cf-PWV. Traditional risk factors including age, BMI, WHR, gender, DM, SBP, and DBP did not correlate with the changes seen. Baseline haemoglobin, corrected calcium, and transferrin was associated with changes in FMD. Baseline transferrin was associated with changes in cf-PWV while baseline phosphate was associated with changes in CCA-IMT.

CKD patients exhibit endothelial dysfunction as measured by FMD [3, 5]. In previous studies, transplantation has been shown to improve FMD, acutely and at 12 months [14, 15]. This recovery may be due to improvements in traditional and uraemia-related non-traditional risk factors [6]. Despite this, FMD values were often still lower compared to controls [5, 6].

CKD patients demonstrate accelerated atherosclerosis as evidenced by high CCA-IMT [3, 6]. The impact of renal transplantation on CCA-IMT is conflicting. One study demonstrated CCA-IMT to progressively increase after 2, 4, and 6 months post-transplantation [16]. Another reported improvements 6 months after transplant [17]. Despite this, values are often still higher compared to the general population [3, 17].

Cf-PWV is a marker of arterial stiffness and predicts the appearance of CVD in CKD, including in transplant patients [11, 18, 19]. Studies evaluating the progression of arterial stiffness over time is conflicting in transplant patients. One study reported no significant change at 12 months after transplantation, while another reported an improvement [19, 20]. Bachelet-Rousseau’s group compared cf-PWV progression in transplant waitlisted patients who were eventually transplanted or were still transplant-pending. No difference in cf-PWV was observed at baseline and upon 1-year follow-up with a short median time of 6.3 (3.8–10.1) months post-transplantation [21]. In contrast, Strozecki’s group showed cf-PWV to progress in transplant patients who were enrolled much later at 36 ± 27 months post-transplantation [22].

Most studies evaluating changes in FMD, CCA-IMT, and cf-PWV in transplant patients do so in immediately post-transplanted subjects. Improvements or non-progression in these parameters shortly after transplantation does not exclude a reversal in recovery. Unlike these studies, our patients were recruited long into the post-transplant period at a median of 86 months post-transplantation. Different pathophysiological mechanisms exist in the development of endothelial dysfunction, accelerated atherosclerosis, and arterial stiffness. Successful kidney transplantation can eliminate important factors that contribute to the progression of these vascular aberrations. However, during long-term follow-up, cardiovascular risk factors often remain and some even worsen, which may explain the progression seen. Additionally, immunosuppression may prevent further improvement and eventual deterioration.

Failure in graft function can explain the progression of endothelial dysfunction in the late post-transplant period. Elimination of ureamic milieu may be particularly important in the restoration of vascular structure and function immediately after transplantation. Interestingly, we found baseline corrected calcium to be associated with changes in FMD and baseline phosphate to be associated with changes in CCA-IMT. Studies have described progressively decreasing FMD with greater renal impairment [3, 5]. Improvements in eGFR after transplantation can explain improvements in FMD in the newly transplanted. However, continued functional decline after kidney transplantation has been noted, studies have reported rates of graft function loss ranging from − 1.90 mL/min/y to − 2.38 mL/min/y in transplant recipients [23–25]. Indeed, our transplant patients demonstrated worsening kidney function at their follow-up visit. However, we could not demonstrate an association between the decline in eGFR and FMD upon follow-up. We too did not demonstrate an association between changes in cf-PWV and eGFR. However, in contrast, associations between PWV and eGFR are inconclusive [26, 27]. eGFR has also been shown to be a significant independent predictor of CCA-IMT [28]. Hence, the restoration in eGFR post-transplant can explain improvements in CCA-IMT. In a study by Yilmaz et al., the improvement in CCA-IMT in their cohort of 178 newly transplanted patients was associated with an accompanying increase in eGFR [17]. Nevertheless, the decline in graft function thereafter can also explain the inevitable progression of CCA-IMT. Yet, again, although we observed parallel changes in eGFR and CCA-IMT in our transplant patients, we did not demonstrate an association between the two. However, consider that the mean eGFR increased from 6.3 ± 4.0 mL/min/1.73 m^2^ to 85.8 ± 13.7 mL/min/1.73 m^2^ in patients from Yilmaz’ group [17]. Whereas we only observed a decrease of 6.06 ± 9.60 mL/min/1.73 m^2^ upon follow-up. Correlations between CCA-IMT and eGFR may only be seen with substantial changes in eGFR.

It is important to consider the role of immunosuppressants in enabling the progression seen. Calcineurin inhibitors increase endothelin levels and abrogate nitric oxide-induced vasodilation, making them potent vasoconstrictors [29, 30]. Additionally, they promote intravascular fibrosis and cause sodium retention leading to hypertension [30]. Hence, these unwanted effects from immunosuppressants may impair endothelial function and promote arterial stiffness in the long run [5, 6].

Another factor that warrants consideration is the dyslipidaemia seen in our transplant patients. Metabolic syndrome is a novel multiplex CVD risk factor that includes dyslipidaemia, central obesity, dysglycaemia, and hypertension. It is common in those with CKD, evidenced by reports of prevalence ranging from 30% in stage 3–4 CKD to 69% in incident haemodialysis patients [31, 32]. Our transplant population had more dyslipidaemics compared to controls. In a cohort of 198 CKD patients, CCA-IMT was found to be closely associated with traditional cardiovascular risk factors, including dyslipidaemia and DM [33].

Anaemia is common in CKD and its aetiology is multifactorial in nature. However, erythropoietin deficiency seems to be the major factor for explaining low haemoglobin in CKD patients. We found that baseline haemoglobin predicted change in FMD in our transplant patients, while baseline transferrin predicted change in FMD and cf-PWV. Indeed, previously it has been reported that haemoglobin is inversely related to FMD in CKD patients with or without diabetes [34, 35]. In contrast, the specific effect of transferrin on PWV and FMD in CKD or kidney transplant patients has not been extensively investigated in the literature.

Ultimately, our study had a small sample size, hence it was difficult to reveal correlations between traditional and non-traditional risk factors with the changes observed, including uraemia, and aspects of metabolic syndrome.

## Conclusion

Our study demonstrates that progressive worsening in surrogate markers of vascular structure and function occur in the post-transplant period upon long-term follow-up. Although transplantation initially alleviates the cardiovascular burden, the vascular disease progresses in the long-term beyond the initial ‘honeymoon’ period. Since cardiovascular mortality is the commonest cause of mortality amongst transplant patients, the nephrology community has long sought for interventions that could improve the adverse cardiovascular picture in such patients. Thus, it is important to further characterise the nature of the changes in vascular abnormalities in kidney transplant recipients, which this pilot study achieves. The ultimate goal of this study is to determine the power needed to demonstrate a difference with an intervention on FMD, cf-PWV, and CCA-IMT in kidney transplant patients.

## Data Availability

The datasets used and/or analysed during the current study are de-identified and available from the corresponding author on reasonable request.

## Abbreviations

ABPI: Ankle-brachial pressure index
BMI: Body mass index
BP: Blood pressure
CCA-IMT: Common carotid artery intima-media thickness
cf-PWV: Carotid-femoral pulse wave velocity
CKD: Chronic kidney disease
CKD-EPI: Chronic kidney disease epidemiology collaboration
CVD: Cardiovascular disease
eGFR: Estimated glomerular filtration rate
FMD: Brachial artery flow-mediated dilation
IQR: Interquartile range
WHR: Waist to hip ratio
